# Polymorphisms within methotrexate pathway genes: Relationship between plasma methotrexate levels, toxicity experienced and outcome in pediatric acute lymphoblastic leukemia 

**DOI:** 10.22038/ijbms.2020.41754.9858

**Published:** 2020-06

**Authors:** Mohammad Ali Esmaili, Ahmad Kazemi, Mohammad Faranoush, Hakan Mellstedt, Farhad Zaker, Majid Safa, Narjes Mehrvar, Mohammad Reza Rezvany

**Affiliations:** 1 Department of Hematology, School of Allied Medical Sciences, Iran University of Medical Sciences, Tehran, Iran; 2 Pediatric Growth and Development Research Center, Institute of Endocrinology and Metabolism, Iran University of Medical Sciences, Tehran, Iran; 3 Mahak Hematology Oncology Research Center (MAHAK-HORC), Mahak Hospital, Shahid Beheshti University of Medical Sciences, Tehran, Iran; 4 Department of Oncology-Pathology, Immune and Gene Therapy Lab, Cancer Center Karolinska (CCK), Karolinska University Hospital Solna and Karolinska Institute, Stockholm 17176, Sweden; 5 Cellular and Molecular Research Center, School of Allied Medicine, Iran University of Medical Sciences, Tehran, Iran; 6 Cellular and Molecular Research Center, Department of Hematology, Faculty of Allied Medicine, Iran University of Medical Sciences, Tehran, Iran; 7 Cancer Research Center, Shahid Beheshti University of Medical Sciences, Tehran, Iran

**Keywords:** ABCB1, ABCG2, Genetic polymorphism, Genotype, Methotrexate, MTHFR, Pediatric acute, lymphoblastic- leukemia, SLC19A1

## Abstract

**Objective(s):**

The current study aimed to investigate the relationship of genetic polymorphism and plasma methotrexate (MTX) levels, toxicity experience and event free survival (EFS) in pediatric acute lymphoblastic leukemia (ALL).

**Materials and Methods:**

The study included 74 ALL patients. Polymerase chain reaction and genotyping of methylene tetrahydrofolate reductase (MTHFR) rs1801133, MTHFR rs1801131, ATP-binding cassette superfamily B1 (ABCB1) rs1045642, ATP-binding cassette superfamily G2 (ABCG2) rs2231142 and solute carrier 19A1 (SLC19A1) rs1051266 genetic variations were performed. The plasma MTX levels were investigated at 48 hr after the first dose of MTX infusion.

**Results:**

MTHFR rs1801133 TT genotype, ABCBa1 rs1045642 CT genotype and ABCG2 rs2231142 CA genotype revealed a statistically significant association with the MTX plasma levels (*P*<0.01, *P*<0.05, *P*<0.05, respectively). The MTHFR rs1801133 TT genotype had a statistically significant association with hematopoietic toxicity (*P*<0.01) and interventions (*P*<0.05). The MTHFR rs1801131 AC genotype was related to the decreased hepatic toxicity (*P*<0.05). The SLC19A1 rs 1051266 GA genotype was related to the increased hepatic toxicity (*P*<0.05). Only the ABCB1 rs1045642 CT and TT genotypes had a statistically significant correlation with EFS (*P*<0.05, *P*<0.05, respectively).

**Conclusion:**

Our findings showed that genetic polymorphism could be associated with plasma MTX levels, toxicity experienced and EFS in Iranian pediatric ALL.

## Introduction

Acute lymphoblastic leukemia (ALL) is the most common malignant disease in childhood. It is characterized by neoplastic proliferation and accumulation of B or T lymphoblast in bone marrow or/and peripheral blood. The rates of the ALL treatment are about 75 to 80%. This could be somewhat different in developed and developing countries, >80% versus <70%. The mainstay of ALL treatment is a 2- to 3-year schedule of multi-agent chemotherapy. Most of different protocols for the treatment of ALL have three phases: induction, consolidation and maintenance. Many cases of resistance to treatment and disease relapse occur in maintenance and consolidation therapies but rarely occur during induction therapy. In addition, prognostic factors, especially age and white blood cells (WBCs), are very important in response to treatment (1-3). 

**Figure 1:**
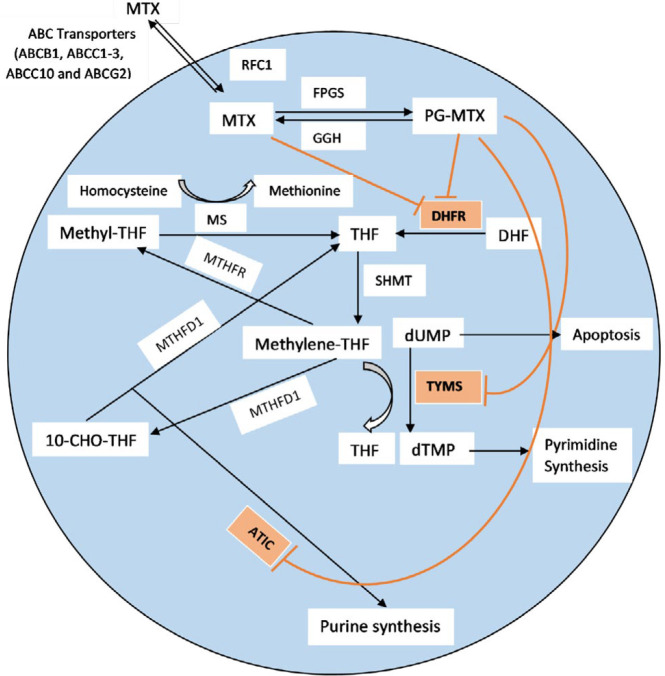
Simplified scheme of cellular pathway and the targets of MTX. MTX enters the cell trough RFC1. After that, MTX inhibits DHFR, TYMS, ATIC. This anti-leukemic drug is pumped from the cell by means of membrane transporters (ABC)

Abbreviations: ABC: ATP-binding cassette; ATIC: 5-aminoimidazole-4-carboxamide ribonucleotide transformylase; DHF: Dihydrofolate; dTMP: deoxythymidine-50-monophosphate; dUMP: deoxyurindine-50-monophosphate Methyl-THF: 5-methyl-tetrahydrofolate; Methylene-THF: 5, 10-methylene-tetrahydrofolate; MTX: Methotrexate; MTX-PG: MTX polyglutamates; MS: Methionine synthase; RFC1: Replication factor C subunit 1; SHMT: Serine hydroxymethyl transferase; THF: Tetrahydrofolate; TS: Thymidylate synthase; TYMS: Thymidylate synthase

Methotrexate (MTX) is one of the major drugs in nearly all treatment protocols for ALL. It is an important anti-folate agent that inhibits enzymes of dihydrofolate reductase (DHFR), thymidylate synthase (TS), 5-aminoimidazole-4-carboxamide ribonucleotide transformylase (ATIC) and has an indirect effect on methylene tetrahydrofolate reductase (MTHFR). MTX, by controlling the *de novo* synthesis of purines and pyrimidines, causes inhibition of cell division in both normal and malignant cells (Figure 1). High-dose methotrexate (HD-MTX), usually defined as a dose > 1000 mg/m^2^, is intravenously administered in consolidation phase of ALL treatment (1, 4-7). MTX administration is influenced by numerous factors such as age, weight, prognostic factors and side effects, as well as host pharmacogenetic and pharmacokinetic parameters. A number of patients do not respond appropriately to HD-MTX therapy. In the other words, HD-MTX often cause toxicity, leading to morbidity and mortality, poor prognosis, interruption and delay in cancer treatment. Thus, identification of these patients is considered a necessity, in order to set up the treatment at the beginning (1, 8-10). 

It is thought that plasma levels and therapy-related toxicity of MTX could be associated with single nucleotide polymorphisms (SNPs) within MTX pathway genes. Examples are variants of MTHFR C677T (rs1801133), MTHFR A1298C (rs1801131), ABCB1 C3435T (rs1045642), ABCG2 C421A (rs2231142) SLC19A1 G80A (rs1051266) and others. MTHFR is an important enzyme in DNA synthesis and DNA methylation. Both SNPs of the MTHFR C677T (rs1801133) and MTHFR A1298C (rs1801131) are accompanied with reduced enzyme activity. Moreover, membrane transporters, the ATP-binding cassette superfamily (ABC) and the solute carrier (SLC) transporters, play an important role in influx and efflux of anti-leukemic agents from cancer cells, respectively. MTX pharmacokinetics is directly associated with these transporters. Some studies have shown that SNPs within MTX pathway genes are related to MTX plasma levels and related side effects. However, obtained findings are not homogenous (11-14). 

Prompted by the abovementioned facts and important effects of SNPs in MTX treatment, the current study aims to investigate the relationship between selected SNPs with plasma MTX levels, toxicity experienced and clinical outcome in pediatric ALL. In addition, this study is the first that has provided data of selected SNPs on the Iranian pediatric population with ALL. 

## Materials and Methods

***Study population and treatment***

Current study was performed in Iran University of Medical Sciences (IUMS), between March 2015 and February 2019. It was a collaboration between IUMS and Mahak Hospital (both are located in Tehran). Mahak is a charity society to support children suffering from cancer. Seventy-four ALL patients were included in this study after signing the informed consent form by patients’ parents. Patients’ peripheral blood specimens were collected from Mahak Hospital in Tehran. The diagnosis of ALL was carried out according to World Health Organization (WHO) criteria (2016) based on morphological findings and immunophenotyping. The eligible criteria were age ≤16-years-old, absence of other active malignancies and complete cytomorphologic remission. 

The SNPs selection criteria were according to following: minor allele frequency (MAF) >5%, genes that were in agreement with Hardy-Weinberg equilibrium (HWE) and finally, strength of evidence of previously published studies. 

The initial treatment was performed according to Berlin Frankfurt Munster (BFM) 2009 protocol. In the BFM 2009 protocol, consolidation regime consisted of administering Cyclophosphamide, Mercaptopurine, high-dose MTX (2000-4000 mg/m^2^/day), Cytarabin, and Lecovorin. All patients received four courses of high-dose MTX every 2 weeks during the consolidation phase of chemotherapy. Administered doses were 2000 mg/m^2^/day for low risk patients and, 4000 mg/m^2^/day for high risk and T cell ALL patients. The method for measurement of the plasma MTX levels was high performance liquid chromatography (HPLC) that was performed in HPLC department of Mahak Hospital (Knaure, Berlin, Germany). 

It was found that evaluation of the plasma MTX levels at 48 hr (with normal level < 1 µmol/l) is independent of treatment protocol and patient’s age. In addition, it reached agreement on that MTX marked effect was only observed at 48 hr. Based on previous studies, the plasma MTX levels were investigated at 48 hr following MTX infusion. Leucovorin rescue was given at 42 hr after initiation of MTX injection at a dose of 15 mg/m^2^ (8, 15, 16). 

The toxic effects of MTX on hematopoietic indices and liver tissue were investigated. Intensity of therapy-related toxicities were evaluated according to the CTCAE v3.0 (Common Terminology Criteria for Adverse Events version 3.0) (17). The highest grade of toxicity observed in each patient during consolidation therapy was registered. MTX toxicities were reported according to a 5-step scoring system (0-V). Hematologic toxicity was determined by the presence of neutrophils <1.5 × 10^9^/l (grade I), <1.5 – 1.0 × 10^9^ /l (grade II), <1 – 0.5 × 10^9^ /l (grade III) and <0.5 × 10^9^ /l (grade IV). Hepatic toxicity was determined by the presence of an increase in Alkaline phosphatase (ALP), and/or Alanine aminotransferase (ALT), and/or Aspartate aminotransferase (AST) > upper limit of normal (ULN) – 2.5 × ULN (grade I), >2.5 – 5.0 × ULN (grade II), >5.0 – 20.0 × ULN (grade III) and >20.0 × ULN (grade IV). For hepatic toxicity, the presence of an increase in bilirubin > ULN – 1.5 × ULN (grade I), >1.5 – 3.0 × ULN (grade II), >3.0 – 10.0 × ULN (grade III) and >10.0 × ULN (grade IV) is also considered (8, 17, 18). 

***DNA extraction and genotyping***

Peripheral blood specimens of patients were collected at 48 hr after MTX injection. After that, genomic DNA was isolated using a genomic DNA isolation kit (Favorgen Biotech Corporation, Taiwan) according to the manufacturer’s instructions. Abovementioned SNPs were identified using the polymerase chain reaction-direct sequencing (PCR-Sequencing). Direct sequencing was performed by Sanger method (Applied Biosystems 3500, CA, USA).

***Statistical analysis***

Deviation from the Hardy-Weinberg equilibrium was assessed using χ^2^. The association between SNPs with plasma MTX levels and therapy-related toxicities were evaluated by Logistic regression. Event free survival (EFS) probabilities were performed by Kaplan-Meier survival curves using the log-rank test and Cox regression analysis. All statistical tests were two-sided. *P-*values ˂ 0.05 were considered statistically significant. SPSS software (version 25.0) and Graph Pad prism software (version 8.0.1) were used for statistical analysis. 

## Results

***Patients and genotyping characteristics***

Of the 74 patients with ALL (aged ≥ 1 to ≤ 16 years), 46 were boys, and 28 were girls (Table 1). Mean and median age of the patients were 6.5 and 5, respectively. Majority of the patients had B-Cell ALL (74.3%). M2-M3 response (≥ 5% blasts) was observed in 14 patients (18.9%). Each patient included in this study received a high dose MTX. Twenty-nine patients (39.2%) had plasma MTX levels more than 1 µmol/l after 48 hr. The number of patients with hematopoietic toxicity was higher compared to those with hepatic toxicity (58.1% vs. 35.1%). Grade V toxicity was not observed in any of patients.

In this study, genotype of five SNPs in genes of MTHFR, SLC19A1, ABCB1 and ABCG2 were detected. The genotype frequencies were in Hardy-Weinberg equilibrium. SNPs, primers and methods used are given in Table 2. In addition, genotype distributions of the MTHFR rs 1801133, MTHFR 1801131, SLC19A1 rs 1051266, ABCB1 rs 1045642 and ABCG2 rs 2231142 gene polymorphisms are outlined in Table 3. 

***Association of SNPs with plasma MTX levels***

To explore the effect of aforementioned SNPs on pharmacokinetics, the relationship between selected SNPs and plasma MTX levels at 48 hr were assessed (Table 4). SNPs of MTHFR rs 1801133, ABCB1 rs 1045642 and ABCG2 rs 2231142 in patients had a statistically significant association with plasma MTX levels at 48 hr. The plasma MTX level at 48 hr was higher in patients with polymorphism of MTHFR rs 1801133 TT genotype when compared to those with CC genotype (OR: 8.654; 95% CI: 1.625-46.078; *P<*0.01). Higher concentrations of plasma MTX were displayed by ABCB1 rs 1045642 polymorphism CT genotype (OR: 4.235; 95% CI: 1.177-15.241; *P<*0.05). Patients with the heterozygote CA genotype had significantly higher plasma MTX levels at 48 hr when compared to the wild type CC genotype for the ABCG2 rs 2231142 gene polymorphism (OR: 4.211; 95% CI: 1.126-15.740; *P<*0.05). 

***Association of SNPs with therapy-related toxicities***

We tested the association between the selected SNPs and grade I-IV therapy-related toxicities among patients. As seen in the Table 5, patients with the heterozygote AC genotype had decreased risk of hepatic toxicity when compared to the wild type AA genotype for the MTHFR rs 1801131 gene polymorphism (OR: 0.313; 95% CI: 0.101-0.969; *P<*0.05). Unlike the MTHFR rs 1801131 polymorphism, heterozygote GA genotype of the SLC19A1 rs 1051266 was associated with increased risk of hepatic toxicity compared to GG genotype (OR: 5.022; 95% CI: 0.995-25.340; *P<*0.05). The MTHFR rs 1801133 TT genotype had a highly significant association with hematopoietic toxicity (OR: 12.353; 95% CI: 1.435-106.344; *P<*0.01) (Table 6). Of note, SNPs of ABCB1 rs 1045642 and ABCG2 rs 2231142 were not significantly related to any toxicity (*P>*0.05).

***Association of SNPs with intervention indicated***

This research is the first study that explored the association between the selected SNPs and interventions indicated. ˝Interventions indicated˝ are defined as lowering the injected MTX dose, discontinuing the MTX drug or hospitalization for hematopoietic and hepatic toxicities (Table 7). Findings showed that only patients with MTHFR rs 1801133 TT genotype had a statistically significant association with those with interventions indicated when compared to CC genotype (OR: 5.778; 95% CI: 1.298-25.709; *P<*0.05). The same trend was observed in patients with the MTHFR rs 1801133 CT genotype, although it was not statistically significant (*P*>0.05). 

***Association of SNPs with EFS***

**Figure 2.**
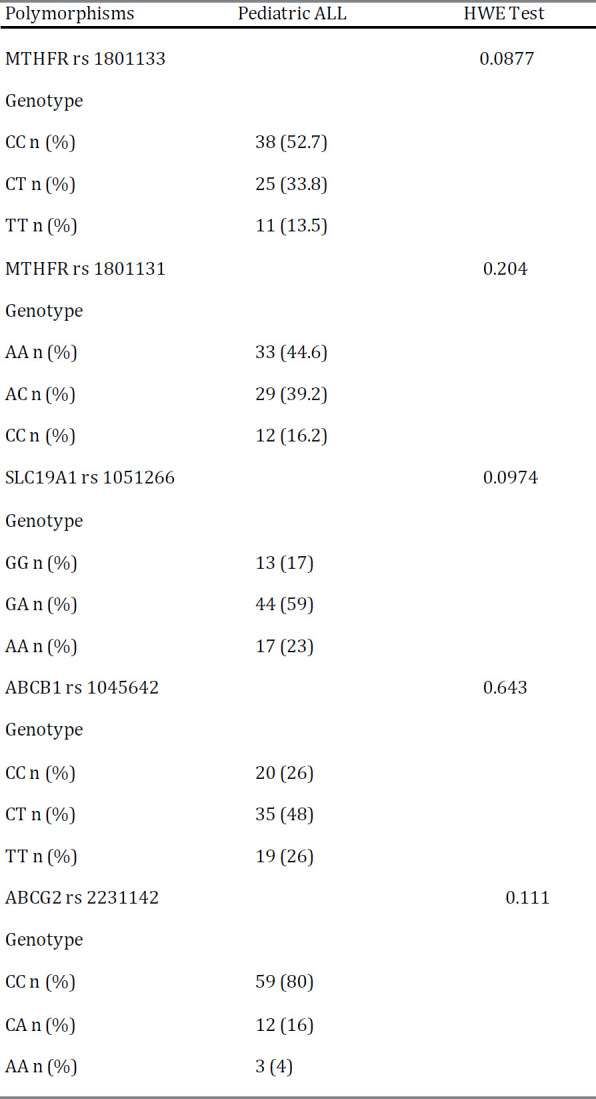
Correlation between ABCB1 rs 1045642 gene polymorphism and EFS. Kaplan-Meier survival curves showed that patients with the ABCB1 rs 1045642 CT and TT genotypes had a poorer EFS than patients harboring CC genotype (*P*<0.05 (A) and *P*<0.05 (B), respectively). *P<0.05

ABCB1: ATP-binding cassette superfamily B1; EFS: Event free survival

**Figure 3:**
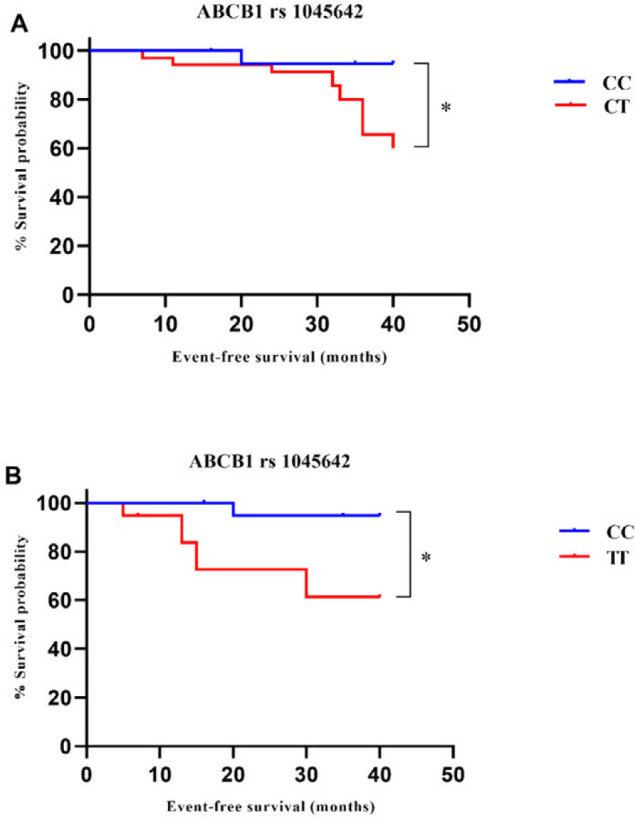
Univariate and multivariate analysis of EFS in patients with pediatric ALL. ALL: Acute lymphoblastic leukemia; EFS: Event free survival; HR: Hazard ratio; CI: Confidence interval; WBC white blood cell. For details, see the text

To investigate the association between the selected SNPs and EFS, patients followed up for 40-month (Figure 2). Kaplan-Meier survival curves showed that the patients with the ABCB1 rs 1045642 CT and TT genotypes had a poorer EFS than patients harboring other genotype (*P<*0.05 and *P<*0.05, respectively), which were confirmed also by univariate Cox regression analysis (*P<*0.05 and *P<*0.05, respectively; Figure 3). There was not a statistically significant association between the ABCB1 rs 1045642 CT genotype and TT genotype (*P*>0.05). 

Finally, multivariate Cox analysis was carried out to investigate the independent prognostic factor of ABCB1 rs 1045642 in pediatric ALL. Independent variables adjusted for patients include age, immunophenotype, WBC count, bone marrow response (day 15) and doses of injected MTX. The result of multivariate Cox regression analysis suggested that ABCB1 rs 1045642 CT and TT genotypes and bone marrow response (day 15) can be used as prognostic factors for the EFS of pediatric ALL (Figure 3). The adjusted hazard ratio was 9.674 (95% CI: 1.187-78.868; *P<*0.05) for patients with the ABCB1 rs 1045642 TT genotype when compared to those with CC genotype. In addition, patients with the ABCB1 rs 1045642 CT genotype had a shorter prognosis compared to those with CC genotype (HR: 8.892 95% CI: 1.168-67.723; *P<*0.05). Bone marrow response (day 15) was also significantly correlated with shorter EFS in patients with ALL (HR: 2.738 95% CI: 1.106-6.783; *P<*0.05). 

## Discussion

In recent decades, tremendous advances in treating pediatric ALL and increasing the survival expectancy of patients have provided. However, there are still clinical concerns. For example, some patients experience adverse events during chemotherapy; in particular, toxicities of HD-MTX are considered as a notable concern (8). These adverse events are associated with poor prognosis, which can be the cause of delay and interruption in cancer treatment. It has been reported that SNPs within MTX pathway genes (pharmacogenomics) have vital role in response to therapy, although some results are obscure (8). These ambiguous findings are mostly due to differences in the genetic pools of populations or studied racial groups (genetic polymorphism), differences in the age group of patients, differences in treatment protocols and different sizes of evaluated groups (8, 10, 12, 18). Meta-analysis review articles can be useful in order to accurate interpretation and conclusion of the reached findings of difference studies. Achieved results from these studies are also conflicting, which will be discussed (23). On the other hand, about 15-20% of patients with ALL will relapse. Gene polymorphisms may play a key role in determining relapse risk and toxicity experienced. Therefore, discovery of new prognostic factors is considered as a necessity in monitoring patients’ response to therapy and predicting their outcome (19). 

***MTX pharmacokinetics***

Here in, we investigated the association between five SNPs within MTX pathway genes consisting of MTHFR rs1801133, MTHFR rs1801131, ABCB1 rs1045642, ABCG2 rs2231142 and SLC19A1 rs1051266, with plasma MTX levels at 48 hr, toxicity of HD-MTX, as well as outcome in 74 Iranian patients with ALL. Of these, MTHFR rs1801133, ABCB1 rs1045642 and ABCG2 rs2231142 SNPs showed a significant association with the MTX plasma levels at 48 hr. For MTHFR rs 1801133 gene polymorphism, plasma MTX level at 48 hr was higher in patients with TT genotype compared to those with CC genotype (Table 4). This finding was in consistent with the previous reports (8, 20). Kantar *et al.* (2009) reported that patients with MTHFR rs 1801133 CT or TT genotype had lower MTX plasma levels at 36 hr , 42 hr and 48 hr, although MTX levels were higher at 24 hr in this group (21). In some studies, it was characterized that there are not a significant association between MTHFR rs1801133 gene polymorphism and MTX plasma levels (9, 14). The current study demonstrated that ABCB1 rs1045642 CT genotype is associated with higher MTX plasma level at 48 hr. This finding was nearly consistent with a study on Malaysian population. In recent study, the ABCB1 rs1045642 TT genotype had a significant association with higher MTX plasma levels at 48 hr (8). Nevertheless, our finding is different to the results of Chinese study (18). This research showed that ABCG2 rs2231142 CA genotype is related to higher MTX plasma levels at 48 hr. This finding is in contrast to the previous studies, which there were not a significant association between ABCG2 rs2231142 gene polymorphism and MTX plasma levels at 48 hr (8, 20). 

***MTX-related toxicities***

This study showed that patients with MTHFR rs1801133 TT genotype were associated with increased hematopoietic toxicity compared to those with CC genotype (Table 6), which is supported by previous reports (8, 15, 22-24). Some studies demonstrated that SNP of MTHFR rs1801133 could be associated with decreased hematopoietic toxicity (21, 25, 26). However, other reports have not mentioned a significant association between MTHFR rs1801133 and any toxicity experienced (11, 14, 27-29). Although several reports described contradictory results, there were not a significant association between the SNPs of MTHFR rs1801131, ABCB1 rs1045642, ABCG2 rs2231142 and SLC19A1 rs1051266 and hematopoietic toxicity in our study, which were in concordance with the results in several other studies (8, 13, 20, 25, 30, 31). For example, an association between SLC19A1 rs1051266 GG genotype and hematopoietic toxicity was claimed by Salazar *et al* (2012) (23). Furthermore, studies have demonstrated contradictory findings concerning associations between the selected SNPs and hepatic toxicity (8, 13, 19, 20, 31). This research revealed that MTHFR rs1801131 and SLC19A1 rs1051266 heterozygote genotypes had a significantly decreased and increased association with hepatic toxicity, respectively. A significantly increased association between SLC19A1 rs1051266 genetic variation and hepatic toxicity was also observed in other studies (8, 20). Zaker et *al.* (2017) have shown that SLC19A1 rs1051266 genetic variation is also a risk factor for MTX hepatotoxicity in consolidation phase (32), which is consistent with our study. In the current study, MTHFR rs1801131 genotype is associated with decrease hepatic toxicity, which is different to the previous reports (15, 28, 33). However, some studies have demonstrated that the MTHFR rs1801131 genetic variation is associated with less toxicity that might indicate a protective role (15, 34-36). 

***Interventions indicated***

Compared to patients with the CC and CT genotypes, those with the MTHFR rs1801133 TT genotype had a highly significant correlation with those that interventions indicated (*P<*0.05), although this association was not significant for patients with the CT genotype *P>*0.05). It seems that the MTHFR rs1801133 TT genotype is associated with different manifestations of MTX toxicity. However, more studies should be performed to confirm the relationship (Table 7). 

***Relationship to EFS***

The prognostic roles of selected SNPs in the current study were also investigated. It was revealed that the ABCB1 rs1045642 gene polymorphism could be considered as an independent prognostic factor. This finding was in contrast to Chinese study (18) in which there was not a significant correlation between ABCB1 rs1045642 polymorphism and EFS. Moreover, it was characterized that SNP of the SLC19A1 rs1051266 was not associated with EFS. These findings were consistent with the outcome of another study (18). The current research did not show an effect of MTHFR rs1801133 and MTHFR rs1801131 gene polymorphisms on EFS. Similarly, these results were obtained in several reports (14, 25, 37, 38). Nevertheless, there are also studies that are not in agreement with our results (23, 39). 

As mentioned above, several meta-analyses studies have shown contradictory results for MTHFR rs1801133 (34, 35, 40-43). Some of these studies were in consistent with our study (34, 35, 40, 41), although some of them were not (42, 43). A meta-analysis was available for the SLC19A1 rs1051266 gene polymorphism that suggests this SNP is not a good marker for MTX-related toxicity in pediatric ALL (44). However, in a recent meta-analysis, a recessive genetic model for the SLC19A1 rs1051266 gene polymorphism was applied. In another systematic review, a statistically significant association between the MTHFR rs1801133 gene polymorphism and overall survival was shown (45). On the other hand, Yao *et al.* (2019) did not find any association between SNPs of the MTHFR rs1801133 and MTHFR rs1801133 with the relapse and overall survival (43).

Several possible limitations can explain these conflicting results, which are also major limitations of the current study. First, each of abovementioned studies had different sample size and HWE that can lead to clinical diversity and variation in their findings. This could lead to developing of false results. Second, different genetic models such as multiplicative, heterozygote, homozygote, dominant, recessive and co-dominant were considered in the studies. This limitation is mainly related to review articles. For example, Yao *et al.* (2019) assumed a dominant genetic model for the MTHFR gene polymorphisms (43) that was different with some other studies (40, 45). It should be noted that only comprehensive comparisons could offer additional details. These comprehensive comparisons can be based on a subgroup analysis by ethnicity, genetic models, age groups and disease type. In a systematic review, the use of different genetic models led to the identification of a significant association between MTHFR gene variations with MTX toxicity (40). Third, host-related factors might be important in developing contradictory findings. These included the host’s own disease status, patient’s homocysteine and folate levels and influence of previous chemotherapy. Forth, study quality is also important. These include differences in dose of injected MTX, definition of toxicity grade, length of follow-up, duration and frequency of therapy and different toxicity grading scales. All of these parameters might influence the accuracy of results. Fifth, studies should also focus on the clinical role of these SNPs, but studies do not often have this feature. Sixth, EFS may depend on the treatment protocol applied and sample size in a study. Thus, the relationship between ABCB1 rs1045642 gene polymorphism and EFS must be investigated based on other treatment protocols of ALL and studies with a larger sample size. 

**Table 1:** The clinicopathological characteristics and toxicity experienced in 74 patients with ALL

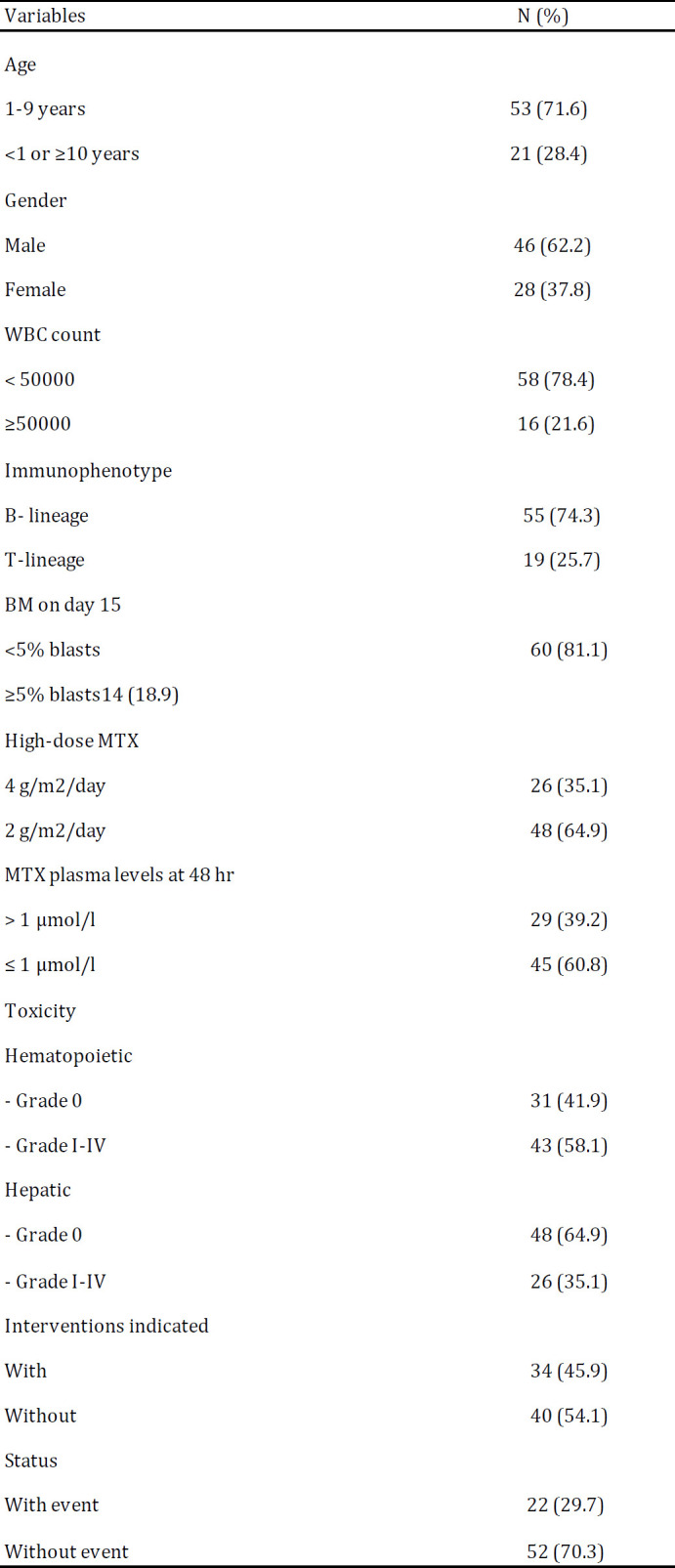

ALL: Acute lymphoblastic leukemia; BM: Bone marrow; MTX: Methotrexate; SD: Standard deviation; WBC: White blood cell

**Table 2:** SNPs included in the study, primers and methods used

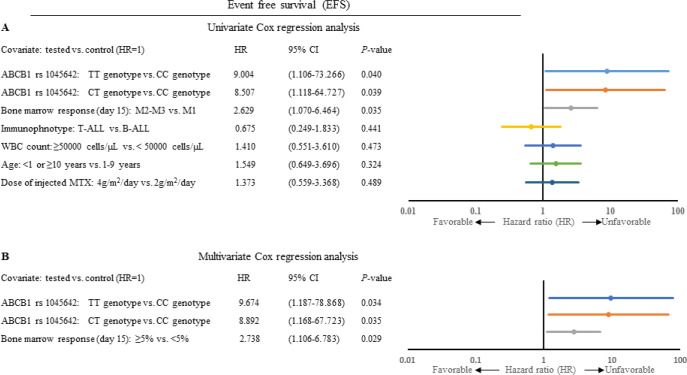

SNP: single nucleotide polymorphism; PCR: polymerase change reaction; F: forward; R: reverse

**Table 3:** Genotype distributions of the MTHFR rs 1801133, MTHFR 1801131, SLC19A1 rs 1051266, ABCB1 rs 1045642 and ABCG2 rs 2231142 gene polymorphisms in pediatric ALL

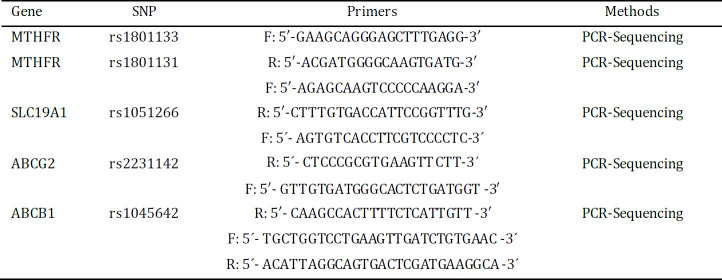

ALL: Acute lymphoblastic leukemia; HWE: Hardy-Weinberg equilibrium

**Table 4:** Logistic regression analysis of association between selected SNPs and MTX plasma levels at 48 hr

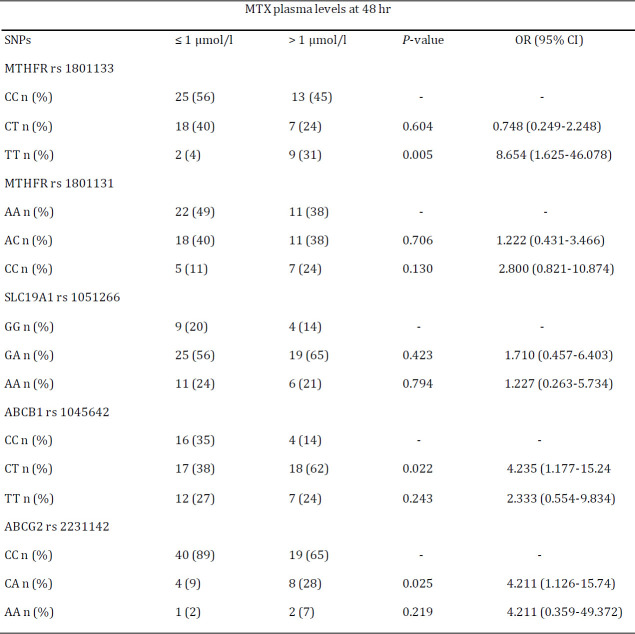

MTX: Methotrexate; SNPs: Single nucleotide polymorphisms; OR: Odd ratio

**Table 5:** Logistic regression analysis of association between selected SNPs with hepatic toxicity (grade 0 vs. grade I-IV)

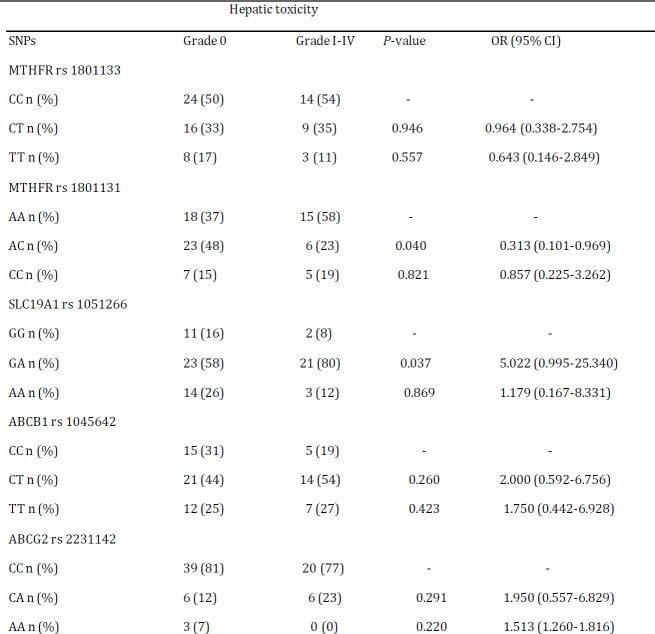

MTX: Methotrexate; SNPs: Single nucleotide polymorphisms; OR: Odd ratio

**Table 6:** Logistic regression analysis of association between selected SNPs with hematopoietic toxicity (grade 0 vs. grade I-IV)

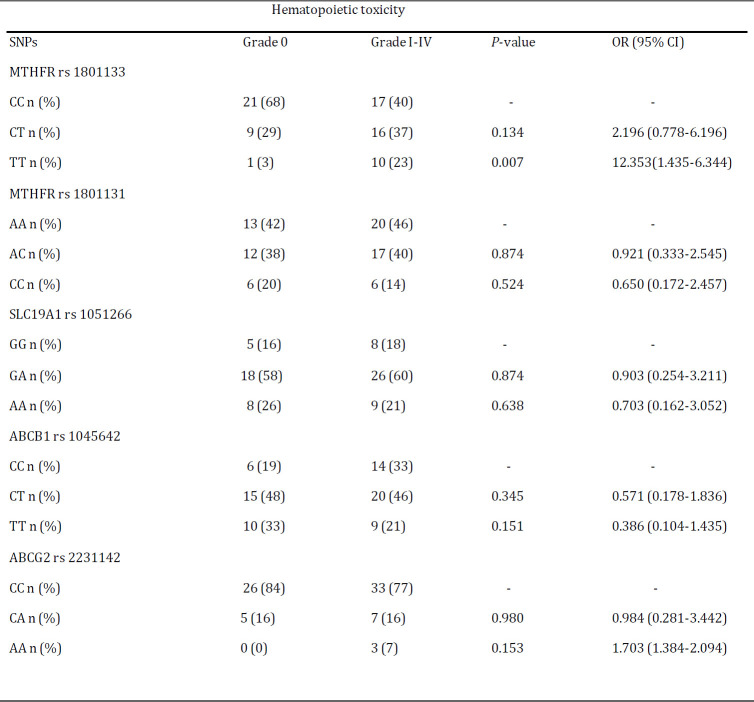

MTX: Methotrexate; SNPs: Single nucleotide polymorphisms; OR: Odd ratio

**Table 7:** Logistic regression analysis of association between selected SNPs with intervention indicated

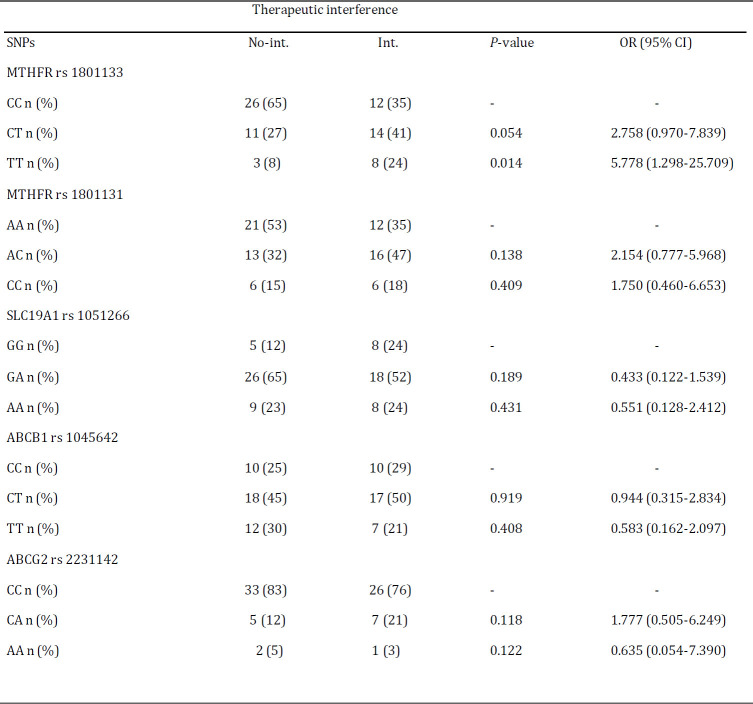

Int.: Intervention; MTX: Methotrexate; SNPs: Single nucleotide polymorphisms; OR: Odd ratio

## Conclusion

These results suggest that genetic polymorphism can be associated with plasma MTX levels, toxicity experienced and outcome in pediatric ALL. Of the selected SNPs, MTHFR rs1801133, ABCB1 rs1045642 and ABCG2 rs2231142 gene polymorphisms revealed a significant association with the MTX plasma levels at 48 hr after MTX administration. In addition, the MTHFR rs1801133 TT genotype was associated with hematopoietic toxicity and interventions indicated. The MTHFR rs1801131 AC genotype was related to the decreased hepatic toxicity, whereas the SLC19A1 rs 1051266 GA genotype was related to the increased hepatic toxicity. Of note, only the ABCB1 rs1045642 gene polymorphism had a significant correlation with EFS. Although, it seems that the MTHFR rs1801133 TT genotype is associated with several manifestations of drug toxicity, further investigations are required to clarify these contradictory results.
